# *In-situ* fabrication of a novel anisotropic PC-matrix light-scattering materials containing spindle-shaped core-shell particles

**DOI:** 10.1038/s41598-019-45030-4

**Published:** 2019-06-17

**Authors:** Yitong Ding, Xingwei Li, Ziru Zhao, Ying Xiong, Shaoyun Guo

**Affiliations:** State Key Laboratory of Polymer Materials Engineering, Polymer Research Institute of Sichuan University, Chengdu, 610065 China

**Keywords:** Materials for optics, Optical materials and structures

## Abstract

Polycarbonate (PC)/Poly(styrene-co-acrylonitrile) (SAN)-organic silica bead (OSB) anisotropic light-scattering materials containing novel spindle-shaped core-shell particles through simple, low-cost hot stretching methods are prepared *in situ*, which have excellent and easily tunable optical properties. The effects of OSB particle size, OSB mass fraction and stretching ratio on the morphology of the spindle-shaped core-shell particles and the scattering properties of PC/SAN-OSB composites were studied in detail. The results show that smaller particle size OSB and smaller draw ratio are more conducive to the production of spindle-shaped core-shell particles. And because of the multiple scattering effects of the spindle-shaped core-shell particles, they have a significant compensation effect on the pattern short-axis light-scattering range of the PC anisotropic materials while ensuring that the pattern long-axis direction light-scattering range is not impaired.

## Introduction

Light-scattering materials^1,2^ are widely used in lighting engineering, display windows, advertising signs, liquid crystal display (LCD) backlight materials, and LED lighting housing materials^3–6^ because they can both transmit light and scatter light. There are many methods for preparing light-scattering materials, such as chemical etching^7^, photofabrication^8^, replication molding^9^, hot embossing^10^, holographic recording^11^, electrospraying^12^, silver halide sensing Gelatin^13^, etc. According to different scattering effects, polymer-matrix light-scattering materials^14–16^ can be classified into isotropic light-scattering materials and anisotropic light-scattering materials. The scattered light distribution of the isotropic light-scattering material is hemispherical, so the scattered light pattern seen in the plane perpendicular to the incident light is circular, and scattering patterns in different directions have the same distribution range. The scattered light of the anisotropic light-scattering material^17^ has a different distribution range in different directions. In practical applications, some occasions have higher light intensity requirements in a single direction, such as road lighting, tunnel lighting, large screen display area etc., and need a certain direction to obtain a larger light-scattering range. Therefore, an anisotropic light-scattering material is prepared to scatter more light in a desired direction, so that the effect of reducing the number of LED light sources and improving the utilization of light energy can be achieved. At the same time, changing the lighting angle by controlling the scattering range reduces and eliminates the effects of glare and pattern on driving and improves driving safety and comfort.

The anisotropic light-scattering materials can be classified into anisotropic surface scattering materials and anisotropic bulk scattering materials depending on the position at which the scattering occurs. An anisotropic surface scattering material is a light-scattering material that uses a surface roughness of a material to obtain a scattering effect. Johnson *et al*.^18^ prepared an anisotropic surface scattering material by electrochemically etching a gallium phosphide wafer in a sulfuric acid solution. Such anisotropic surface scattering materials have simple preparation methods, uniform scattering patterns and high light transmittance, but also have some defects, such as the surface being easily worn and impairing its light scattering properties. On the contrary, the anisotropic bulk scattering material has attracted more and more attention due to its excellent comprehensive performance such as simple preparation method, good use stability, easy mass production and so on. Many researchers have prepared anisotropic light-scattering materials by adding anisotropic scattering particles to the polymer matrix^19^. For example, Takamitsu *et al*.^20^ prepared a scattering polarizer by adding calcium carbonate whiskers to a polyolefin polymer. However, the addition of such an anisotropic scatterer greatly reduces the light transmittance of the material and causes a loss of light.

In previous studies, we prepared polycarbonate (PC)/poly(styrene-co-acrylonitrile) (SAN) and polymethyl methacrylate (PMMA)/polyethylene terephthalate (PET) light-scattering materials by a simple melt blending process, which has outstanding advantages of high light transmittance and high haze^14,21^. Since the viscosity of SAN is lower than that of PC, SAN can be completely oriented during hot stretching, so we have used this feature to prepare PC/SAN anisotropic light scattering materials^22^. Compared with conventional scattering particles, the degree of anisotropy of the light-scattering material can be controlled by the stretching ratio, and it has the advantages of environmental stability, low cost, and high production efficiency. However, as the scattering range in the pattern long-axis direction increases, the scattering range in the pattern short-axis direction decreases greatly, which limits the flexibility of adjusting the scattering range.

In recent years, the particles of the core-shell structure have attracted more and more attention because of their special functions^23,24^. If they are introduced into the light-scattering materials, since they can produce multiple scattering effects^25–27^, the light-scattering ability of the light-scattering materials can be greatly improved. W. Suthabanditpong *et al*.^28^ introduced hollow silica nanoparticles into light-scattering materials, which greatly improved the light-scattering ability of light-scattering materials. It is well known that the selective distribution of non-deformable particles in a polymer blend is determined by the wetting coefficient^29,30^. That is, the non-deformable particles can be selectively distributed among the polymer components which are more compatible therewith. Moreover, the PC^31^ and SAN^32^ can be melt-blended to form a sea-island structure, the SAN is a spherical island phase, and the PC is a marine phase. Therefore, it is possible to prepare a scatterer with a core-shell structure with a SAN as a shell by selecting a non-deformable particle that is more compatible with the SAN. In this paper, PC is used as the matrix, SAN is the scatterer, and OSB (Polymethylsilsesquioxane, which has good thermal stability.) is selectively distributed in the SAN phase by a simple melt blending method to make the core-shell structure scatterer. During the hot stretching at 190 °C, the SAN will be oriented in the direction of stretching, while the OSB does not change due to the higher heat distortion temperature, and the spherical core-shell structure particles are deformed into spindle-shaped core-shell particles. The oriented SAN can provide anisotropic light-scattering capability for the light-scattering material, and the undeformed OSB can complement the loss of light-scattering of the pattern in the pattern short-axis direction. The effects of OSB particle size, OSB mass fraction and stretching ratio on the morphology of novel spindle-shaped core-shell particles and the properties of PC matrix blends were studied in detail.

## Experiments

### Materials

The characteristics of the raw materials used in this work are shown in Table 1. The PC used in this study is a bisphenol A polycarbonate. SAN is a styrene-acrylonitrile random copolymer containing 25% acrylonitrile. OSB is a polymethylsilsesquioxane.

**Table 1:** Raw Materials Used in this Study (^*a*^Determined here by GPC).

Polymer	Source	Commericaldescription	Molecular weight, ${\bar{{\boldsymbol{M}}}}_{{\boldsymbol{w}}}^{{\boldsymbol{a}}}$	Molecular weight distribution	Density(g/cm^3^)	Refractive index	Average diameter(µm)
PC	LG DOW Polycarbonate Ltd.	1201-22	31,809	2.253	1.20	1.586	—
SAN	Formosa Plastic(Ningbo) Ltd.	NF-2200	87,254	1.656	1.06	1.567	—
OSB1	UNICONE Materials Technology (Shanghai) Co.	El1000	—	—	1.3	1.43	10.0
OSB2	UNICONE Materials Technology (Shanghai) Co.	El201	—	—	1.3	1.43	2.0

The PC, SAN, and OSB were dried under vacuum at 80 °C for 12 hours to remove moisture. A torque rheometer (Model RM-200C Torque Rheometer, Harbin Harp Co., Ltd.) was used at 240 °C at a speed of 30 r/min to refine 8 min. The milled samples were molded at 240 °C into a sample of 100.0 mm (length) × 30.0 mm (width) × 1 mm (thickness). Then, the sample was fixed in a high-temperature universal tensile testing machine (CMT-4104), heated to 190 °C, held for 2 min, and thermally stretched at a speed of 60 mm/min, after which the sample was rapidly cooled to fix the orientation. The distance between clamps before stretching is *L*_0_, the distance between clamps after stretching is *L*, and the stretching ratio R is equal to $(L-{L}_{0})/{L}_{0}$. The different specimen compositions are shown in Table 2. Among them, the quality ratio of PC and SAN is 7:3.

**Table 2:** Constituents of the PC/SAN-OSB Blends (x is 0, 0.5, 1.0, 1.5, 2.0, representing OSB mass fraction, 0 wt%, 0.5 wt%, 1.0 wt%, 1.5 wt% and 2.0 wt%, respectively).

Label	Stretch ratio				
	0	1.75	2	2.25	2.5
PC/SAN-OSB1-x	1-0-x	1-1-x	1-2-x	1-3-x	1-4-x
PC/SAN-OSB2-x	2-0-x	2-1-x	2-2-x	2-3-x	2-4-x

### Characterization techniques

The compatibility between OSB and polymer (PC or SAN) is judged by the extent to which OSB affects the glass transition temperature (T_g_) of PC and SAN. The glass transition temperature (T_g_) was measured with a differential scanning calorimeter (TA-Q20). First, the sample was held under a nitrogen atmosphere at 220 °C for 5 minutes to remove moisture and heat history, then cooled to 40 °C and finally reheated to 220 °C. The heating rate is 10 °C/min.

RHEOGRAPH 2002 high pressure capillary rheometer (Germany Gottfert company) is used to determine the rheological properties of the materials. The capillary length to diameter ratio is 30: 1 and we can ignore the export effect. The test shear rate range is 20~2000 s^−1^, processing temperature is 240 °C, preheating time is 4 min.

The microscopic morphology of the samples was observed by scanning electron microscopy (SEM, JSM5900LV, Japan Electronics Co., Ltd.) and transmission electron microscopy (TEM, FEI Tecnai G2 F20, USA.). Anisotropic light-scattering materials were prepared by a method of hot stretching, and after 5 hours of freezing in liquid nitrogen, brittle fractures were carried out in the directions of orientation and vertical orientation, respectively. The gold/palladium alloy layers were applied to the cross section of the sample and the cross section was observed by SEM. For the TEM investigations, the samples were first sliced at room temperature by an ultramicrotome, then stained in steam of an aqueous solution of ruthenium-tetroxide (RnO_4_) for 30 min, and finally observed by electron microscopy.

**Figure 1:**
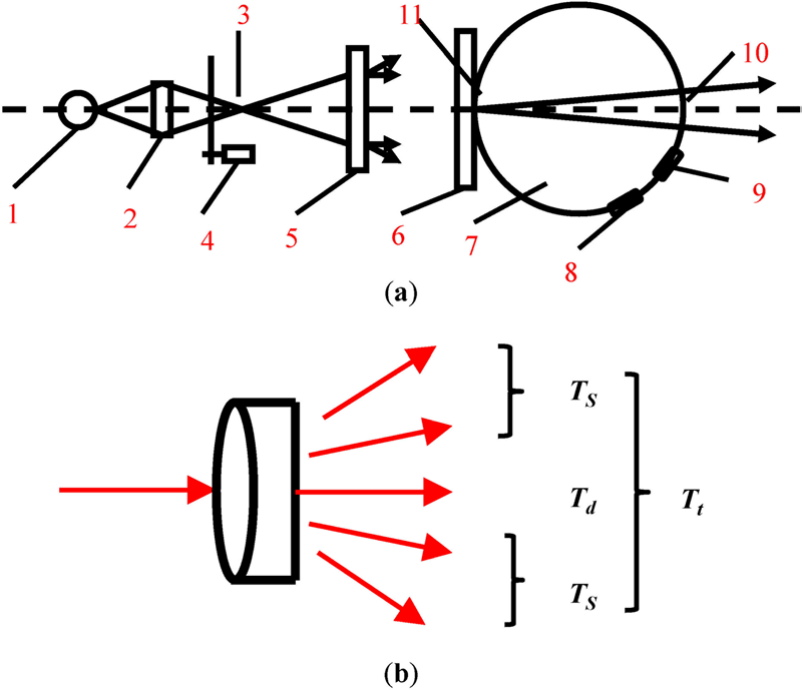
(**a**) Optical system of the Transmittance-Haze testing instrument: (1) source (2) condenser (3) aperture (4) modulator (5) lens (6) sample (7) integrating sphere (8) photocell (9) reflectance standard (10) emergent window (11) entrance window. (**b**) The diagram of direct transmittance (T_d_), scattered transmittance (T_s_) and transmittance (T_t_).

As shown in Fig. 1(a), transmittance (*T*_*t*_) and haze (*H*) of the samples were measured by Transmittance-Haze test instrument (WGT-S), according to ASTM D1003-61, which is made by Shanghai Precision and Scientific Instrument Corporation, China. *T*_*t*_ and *H* are expressed as follows:

1 $${T}_{{\rm{t}}}=\frac{{I}_{{\rm{t}}}}{{I}_{0}}$$

2 $$H=\frac{{({I}_{t})}_{2.5}^{90}}{{I}_{t}}$$

where *I*_*t*_ means the total transmitting light intensity, *I*_*0*_ means the incident light intensity, and ${({{\rm{I}}}_{{\rm{t}}})}_{90}^{2.5}$ is the forward transmitted light intensity of the scattering portion exceeding 2.5°.

As shown in Fig. 1(b), the direct transmission (*T*_*d*_) of the samples were measured using an ultraviolet-visible spectrophotometer (UV-1750, Shimadzu, Japan). *T*_*d*_ is expressed as follows:

3 $${T}_{d}=\frac{{I}_{d}}{{I}_{0}}$$

where *I*_*0*_ is the incident light intensity, *I*_*d*_ is the direct light intensity.

**Figure 2:**
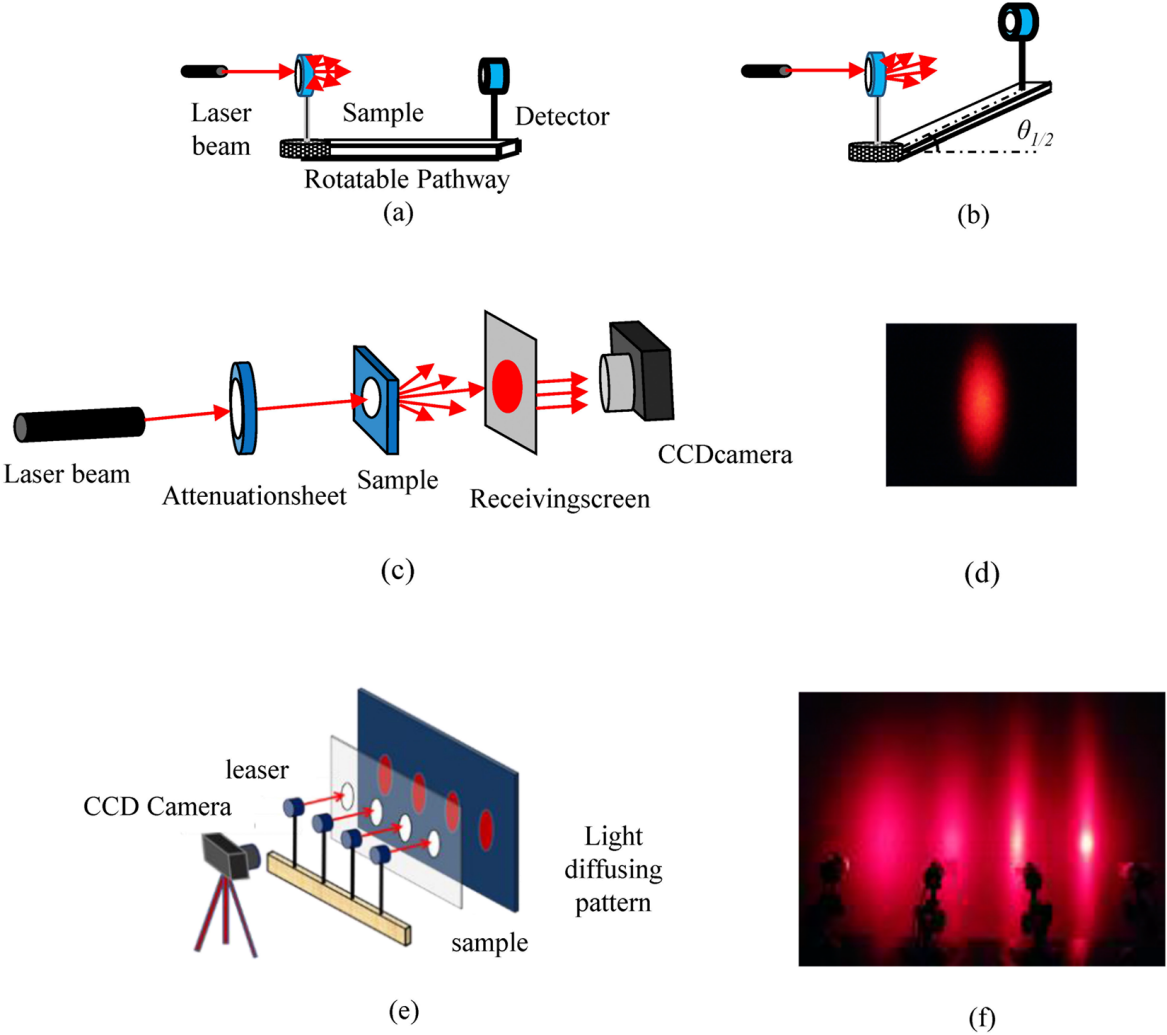
(**a**) The diagram of the scattering angle testing setup. (**b**) Schematic diagram of seeking out the half angle. (**c**) The diagram of the diffusing pattern measuring setup. (**d**) The example of a diffusing pattern. (**e**) The diagram of multi-source scattering pattern test platform and (**f**) the example of multi-source light-scattering patterns.

The scattering angle is defined as the angular difference corresponding to half the peak of the scattered light intensity^33,34^. Since the scattered light intensity distribution is a symmetric Gaussian distribution with symmetry on both sides, the scattering angle can be calculated from 2*θ*_1/2_. The self-built testing setup which is used to measure the intensity of scattered light is shown in Fig. 2(a,b). The light source is a He-Ne laser with a wavelength of 632.8 nm. The sample was fixed on a rotating center with an angle scale. The other side of the orbit was an optical power meter (SZX optical power meter), 485 mm away from the sample. First turn the track to find the maximum value of the scattered light intensity, where the value of the angle scale is *θ*_0_; then slowly turn the track, when the scattered light intensity reaches half the peak, and record the corresponding angle *θ*, *θ*_1/2_ = |*θ* − *θ*_0_|, which is the measured scattering angle size.

The diffusing patterns were characterized by a home-built instrument (shown in Fig. 2(c)). The light source is a He-Ne laser. In a darkroom, the scattered light spot projected under the screen was recorded with a CCD camera. The resulting scattering patterns are shown in Fig. 2(d).

The scattering angles in both directions will change at the same time with the change of OSB mass fraction, OSB particle size and draw ratio. Therefore, the multi-light source characterization method can be used to investigate the change of the scattering range in both directions. He-Ne laser was chosen as the light source, which was fixed in parallel with the light source spacing of 105 mm, the sample holder was placed in parallel in front of the light source, the scattered light patterns were projected on the white receiving screen, and the CCD camera was used to capture the scattered light patterns in the dark environment. A home-built platform for characterizing multi-light scattering effects is shown in Fig. 2(e) and the example of multi-source light-scattering pattern is shown in Fig. 2(f).

## Results and Discussion

### Microstructure and scattering mechanism

**Figure 3:**
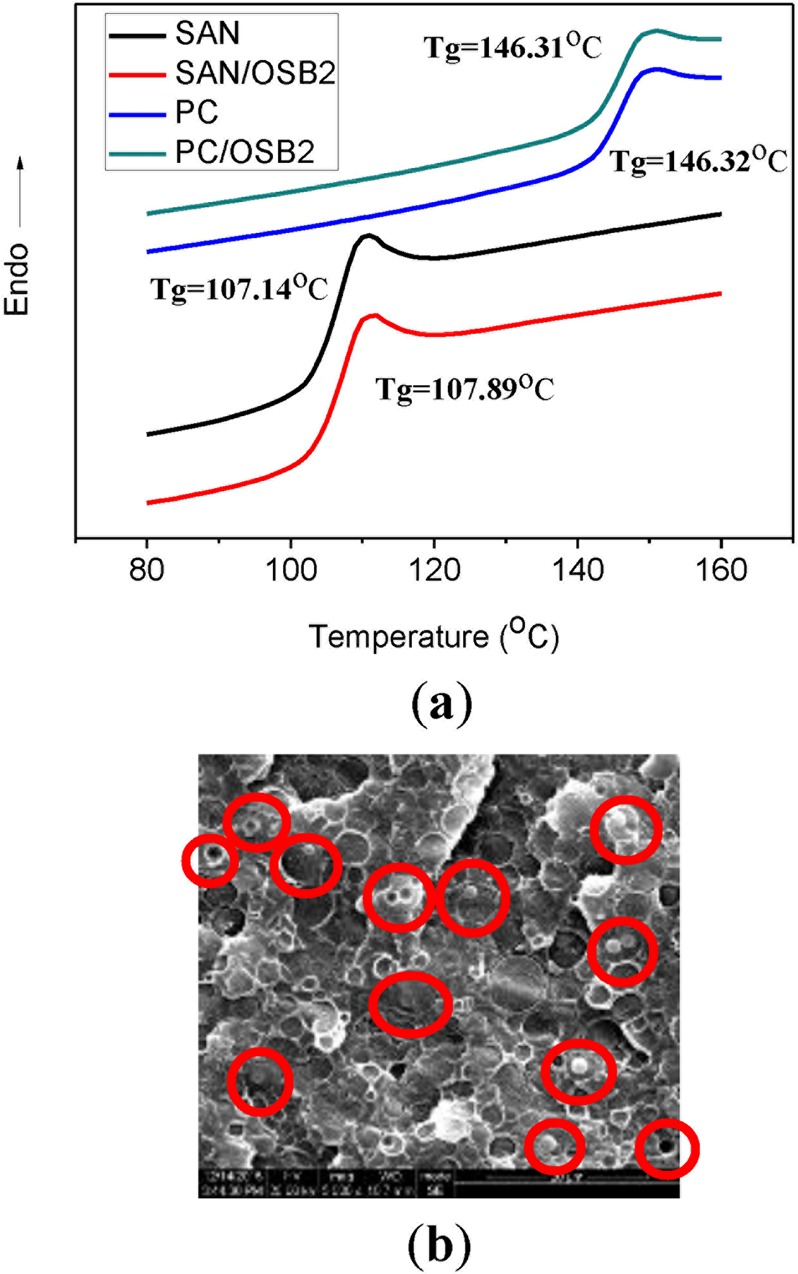
(**a**) DSC curves of pure polymers and their blends with OSB. (**b**) SEM micrographs of 2-0-1.0 sample.

The compatibility between OSB and PC or SAN can be judged by the influence of OSB on the T_g_ of PC or SAN. As shown in Fig. 3(a), when 1% of OSB was added, the T_g_ of the SAN increased from 107.14 °C to 107.89 °C, while having little effect on the T_g_ of the PC, indicating a better compatibility between the OSB and the SAN. This can be more clearly demonstrated by the result of scanning electron microscopy. Figure 3(b) is a fragile cross-sectional view of a 2-0-1.0 sample. It can be clearly seen from the figure that the OSB particles are selectively distributed in the SAN phase and distributed together with the SAN in a core-like shell structure in the PC matrix^35^.

**Figure 4:**
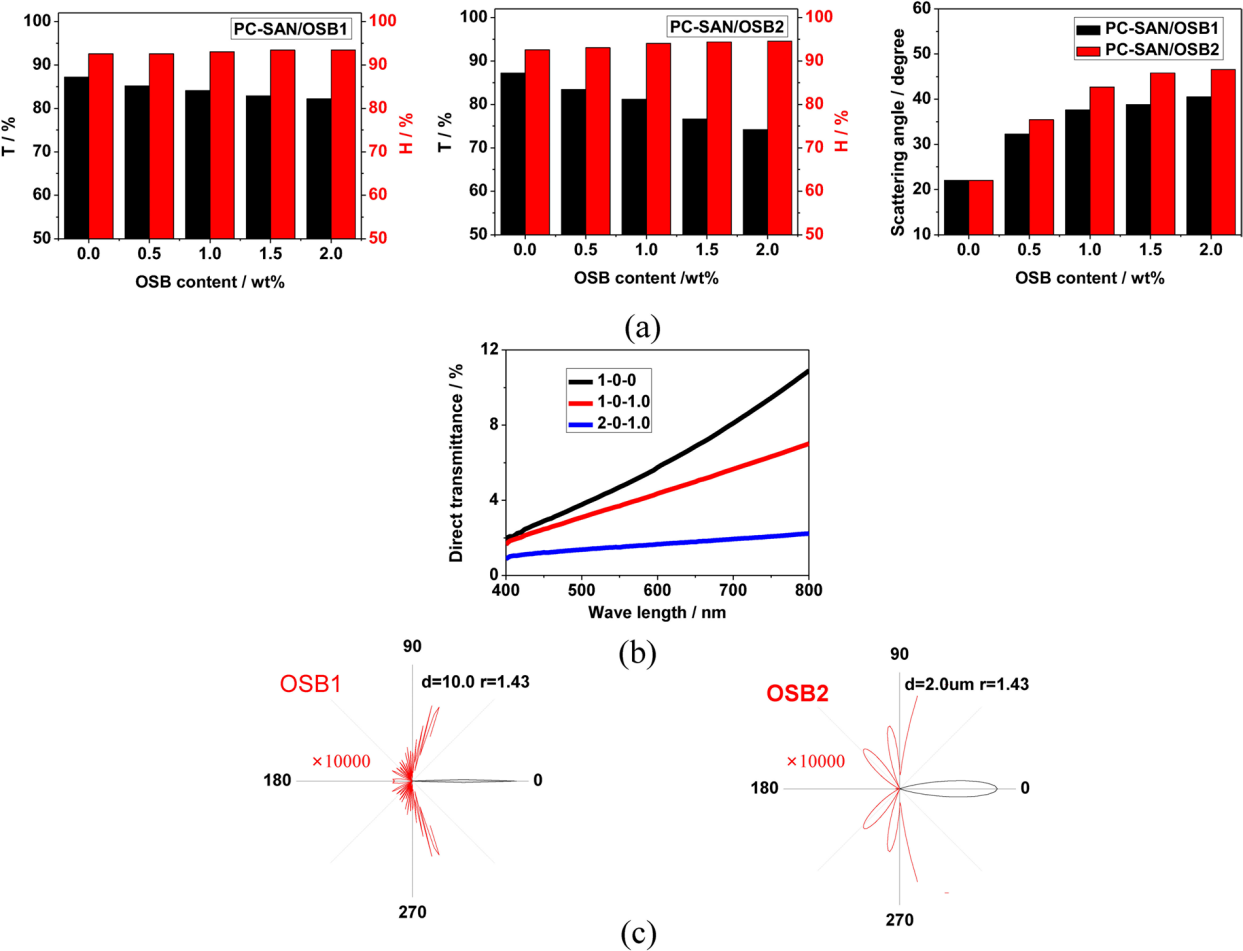
(**a**) The haze, transmittance and scattering angles of two groups with increasing content of OSB. (**b**) UV-Vis spectrum of samples. (**c**) Calculated scattering profiles of single particle with different size using Mie scattering theory.

The optical properties of PC-based light scattering materials containing SAN-OSB core-shell structured complex scattering particles were investigated in detail. Figure 4(a) shows the effect of core-OSB particle content on light transmission, haze and scattering angle. It can be seen that the addition of OSB particles is beneficial to maintain the high haze of the PC-based light scattering material and greatly increase its light scattering angle, but it will slightly reduce its light transmittance. When the content of OSB2 particles is increased to 1.0%, the light transmittance is 81.2%, the haze is 94.06%, and the light scattering angle is 42.68°, which is a light diffusing material excellent in light scattering properties. Moreover, the addition of OSB particles greatly reduces the direct transmittance of PC/SAN light-scattering materials, especially for OSB2 particles, which has a stronger effect on the reduction of direct light transmittance (Fig. 4(b)). The direct transmittance of visible light waves is reduced by 100–400% (550 nm, 214%), which is very helpful in hiding LED lights and eliminating glare from LED lights.

When $1\le \alpha  < 40$ (α is the size parameter), that is, the particle size of the scattering particles is equivalent to the wavelength of the incident light, the light scattering phenomenon of the particles is applied to the Mie scattering theory. In 1908, Mie set up the Mie scattering theory from the Maxwell equations for electromagnetic waves as follows:

4 $$\alpha ={n}_{2}\pi d/\lambda $$

5 $$I=\frac{{\lambda }^{2}}{8{\pi }^{2}{r}^{2}}{I}_{0}({i}_{1}+{i}_{2})$$

Among them,

$$\begin{array}{rcl}{i}_{1} & = & {s}_{1}(m,\theta ,\alpha )\times {s}_{1}^{\ast }(m,\theta ,\alpha )\\ {i}_{2} & = & {s}_{2}(m,\theta ,\alpha )\times {s}_{2}^{\ast }(m,\theta ,\alpha )\\ {s}_{1} & = & \sum _{n=1}^{\infty }\frac{2n+1}{n(n+1)}({a}_{n}{\pi }_{n}+{b}_{n}{\tau }_{n})\\ {s}_{2} & = & \sum _{n=1}^{\infty }\frac{2n+1}{n(n+1)}({a}_{n}{\tau }_{n}+{b}_{n}{\pi }_{n})\\ {a}_{n} & = & \frac{{\phi }_{n}(\alpha ){\phi }^{^{\prime} }(m\alpha )-m{\phi }_{n}^{^{\prime} }(\alpha ){\phi }_{n}(m\alpha )}{{\zeta }_{n}(\alpha ){\phi }_{n}^{^{\prime} }(m\alpha )-m{\zeta }_{n}^{^{\prime} }(\alpha ){\phi }_{n}(m\alpha )}\\ {b}_{n} & = & \frac{m{\phi }_{n}(\alpha ){\phi }_{n}^{^{\prime} }(m\alpha )-{\phi }_{n}^{^{\prime} }(\alpha ){\phi }_{n}(m\alpha )}{m{\zeta }_{n}(\alpha ){\phi }_{n}^{^{\prime} }(m\alpha )-{\zeta }_{n}^{^{\prime} }(\alpha ){\phi }_{n}(m\alpha )}\\ {\pi }_{n} & = & \frac{d{P}_{n}(\cos \,\theta )}{d(\cos \,\theta )}\\ {\tau }_{n} & = & \frac{d}{d\theta }{P}_{n}^{(1)}(\cos \,\theta )\\ {\phi }_{n}(z) & = & {(\frac{z\pi }{2})}^{1/2}{J}_{n+\tfrac{1}{2}}(z)\\ {\zeta }_{n}(z) & = & {(\frac{z\pi }{2})}^{1/2}{H}_{n+\tfrac{1}{2}}^{(2)}(z)\\ m & = & {n}_{2}/{n}_{1}\end{array}$$

where *m* is the relative refractive index between particle (*n*_*2*_) and matrix (*n*_*1*_), *d* is the diameter of the sphere, *λ* is the incident wavelength, *I* is scattering intensity(watt/m^2^), *r* is the distance from the center of the sphere, *I*_*0*_ is the incident light intensity (watt/m^2^), *θ*_*0*_ is the scattering angle, ${\phi }_{n}(z)$ and ${\zeta }_{n}(z)$ are the Riccati–Bessel functions, and ${P}_{n}(\cos \,\theta )$, ${P}_{n}^{(1)}(\cos \,\theta )$ are the Legendre and associated Legendre functions of cos *θ*, respectively^5^.

According to Mie scattering theory, forward (the ray from 0° to 90° and from 270° to 360° (0°)) and backscattering (the ray from 90° to 180° and from 180° to 270°) occurs after light passes through a single silicone particle having a different refractive index than the medium. Figure 4(c) is scattering profiles of light passing through the OSB dispersed in the SAN phase, calculated according to the Mie scattering theory (The red line indicates enlarged 10000 times, and the refractive index of the SAN serves as the refractive index of the substrate, assuming that the wavelength of the incident light is 550 nm.). The particle size of OSB has a great relationship with the light scattering effect. The backscattering effect of silicone particles with a particle size of 2μm is stronger and the range of forward scattering is larger. This is one of the reasons why the PC-SAN/OSB2 sample has a lower transmittance and a larger range of light scattering. In addition, the same mass of OSB particles, the number of particles having a smaller particle size is more, so a larger scattering area is produced. This is another important reason why the PC-SAN/OSB2 sample has a lower transmittance and a larger range of light scattering.

**Figure 5:**
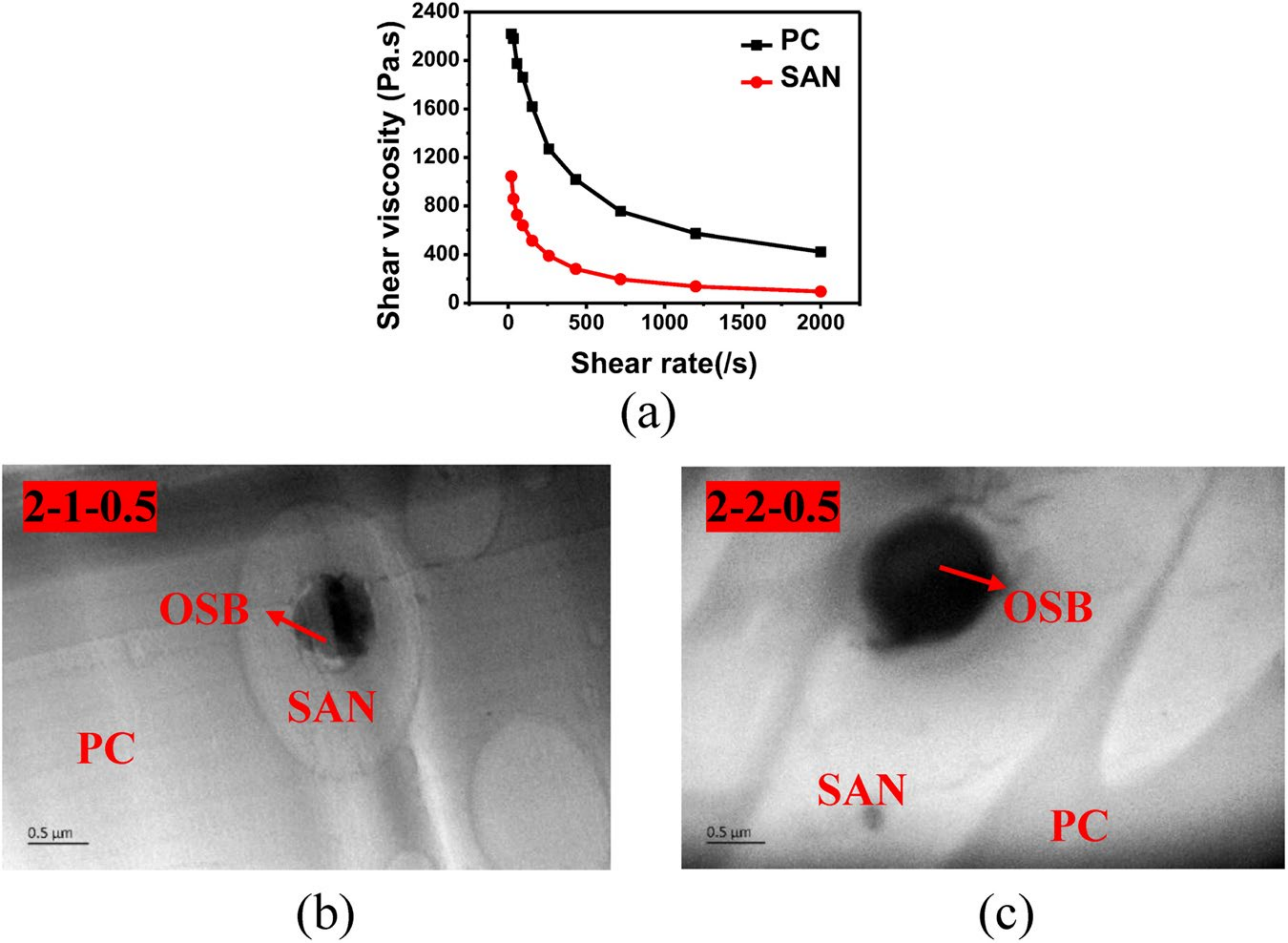
(**a**) Melt viscosity versus shear rate of PC and SAN at 240 °C. (**b**,**c**) TEM images of 2-1-0.5 and 2-2-0.5.

From the above results, it is understood that the PC-based material containing the SAN-OSB-based core-shell structure has excellent optical properties. And it is known that PC and SAN are partially compatible blends, and SAN is spherically dispersed in the PC matrix in the absence of stretching. The glass transition temperatures of PC and SAN were 146 °C and 107 °C, respectively. When stretched at 190 °C, both the orientation of SAN and PC occurred. Figure 5(a) is a graph of the melt shear viscosity of a PC, SAN, as a function of shear rate for a high-pressure capillary test at 240 °C. As the shear rate increases, both polymers undergo shear thinning, but at all shear rates (when the shear rate is 100 s^−1^, ${\eta }_{PC}/{\eta }_{SAN}=1861/639\approx 3/1$). According to Delaby’s theory^36^, SAN in the PC matrix easily deformed. Therefore, we can use this feature to prepare anisotropic PC-based light scattering materials with SAN as a scatterer.

**Figure 6:**
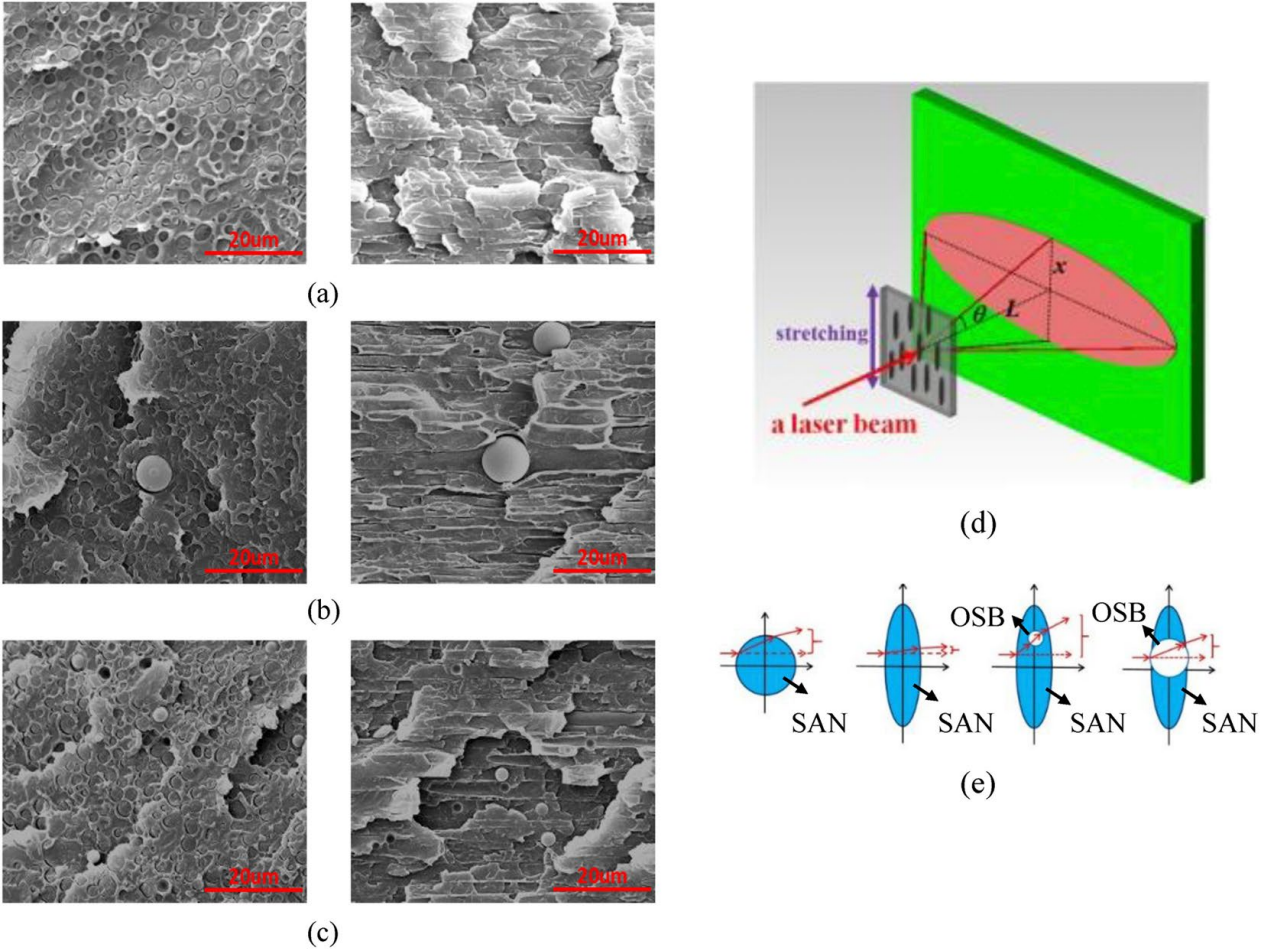
(**a**) The SEM morphology of PC/SAN, the draw ratio is 2. (**b**) and (**c**) The SEM morphologies of 1-2-1.0 and 2-2-1.0, respectively. (**d**) and (**e**) Schematic diagram of light-scattering materials and light-scattering particles with the spindle-shaped core-shell structure.

As shown in Fig. 5(b,c), the TEM images of PC/SAN-OSB2 after hot drawing, the SAN is oriented and deformed after hot stretching. And it is dispersed as a long spindle-shaped island structure in the PC matrix. However, the OSB particles do not change during the stretching process because of it’s high thermal deformation temperature. Therefore, the spherical SAN-OSB core-shell structure particles are deformed into spindle-shaped core-shell particles. And as the draw ratio increases, the aspect ratio of the SAN phase gradually increases. Figure 6(a–c) are horizontal and vertical SEM images of PC/SAN, PC/SAN-OSB1, PC/SAN-OSB2 samples after hot drawing. When the OSB particle diameter is smaller than the oriented SAN short axis diameter, the OSB particles are completely distributed in the SAN (Fig. 6(c)). When the OSB particle diameter is larger than the SAN short axis diameter, the OSB will be partially distributed in the SAN phase and protruded out of the SAN phase on both sides (Fig. 6(b)).

The stretched PC/SAN-OSB samples are known to have the following micrographs: SAN is long spindle-shaped, OSB is completely distributed in SAN phase or partially in SAN phase. As shown in Fig. 6(d), the stretching direction of the anisotropic light-scattering material is perpendicular to the long-axis scattering direction of the scattered light pattern. As can be seen from the figure, the anisotropic scattered light pattern has a significant loss of scattered light in the pattern short-axis scattering direction. As shown in Fig. 6(e), spindle-shaped core-shell particles can produce multiple scattering effects to compensate for these losses. Moreover, if the core (OSB) particle size is too large, the spindle-shaped core-shell structure will be destroyed, and the multiple scattering effect will be weakened, then the compensation effect of the nuclear (OSB) on the pattern short-axis scattering direction of the scattered pattern will be correspondingly weakened.

### Effect of OSB particle size and mass fraction on scattering angle at a certain draw ratio

**Figure 7:**
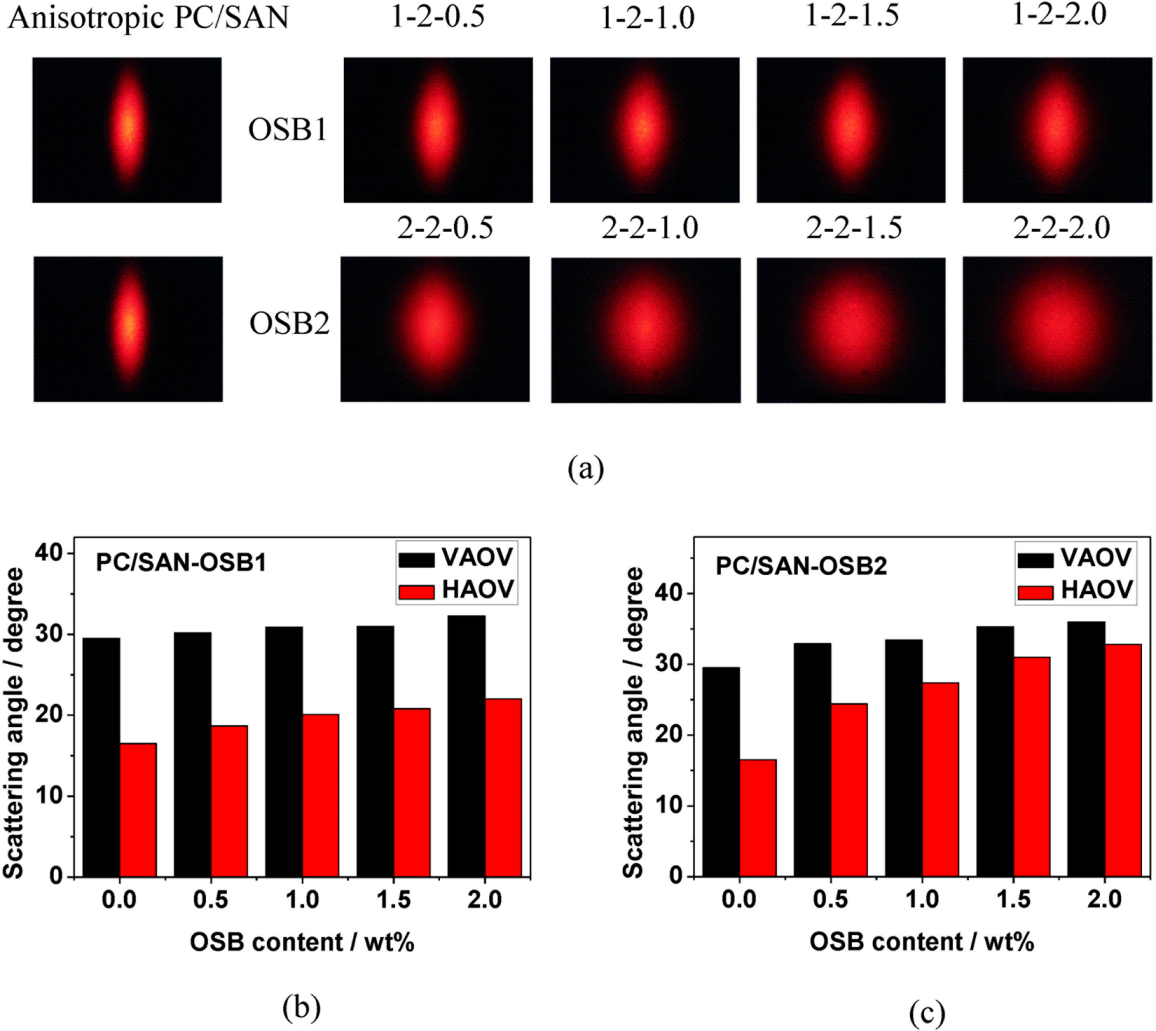
(**a**) Scattering patterns of two groups with OSB mass fraction increasing from 0 to 2.0 wt%, the draw ratio is 2. (**b**) and (**c**) Scattering angle of two groups with OSB mass fraction increasing from 0 to 2.0 wt%.

Figure 7(a) shows a scattering pattern of two groups of OSB mass fraction of 0.5 wt% to 2.0 wt% when the draw ratio is 2 in the PC/SAN-OSB system. When the mass fraction of OSB is 0 wt%, the elliptical scattered light pattern is obtained. The scattering range in the pattern short-axis direction is obviously smaller than that in the pattern long-axis direction, and the center of the pattern has a yellow area with poor shielding property. After OSB is added, the center brightness decreases and the scattering range in the pattern short-axis direction is increased. The difference is that, after adding OSB1, the scattered light pattern can always maintain a more pronounced ellipse, that is, the scattered light of the sample can maintain good anisotropy. As the mass fraction of OSB1 increases, the pattern short-axis direction scattering range of the sample increases at a slower speed, and the pattern long-axis direction scattering range of the sample has a little change. After OSB2 is added, the pattern short-axis direction scattering range of the sample increased rapidly with the increase of OSB2 mass fraction. The pattern long-axis direction scattering range of the sample does not change much. Figure 7(b,c) show the change of scattering angle of two groups with increasing OSB mass fraction when the stretching ratio is 2, respectively. With the increase of OSB mass fraction, the VAOV (scattering angle in the pattern long-axis direction) of two groups of samples increased less. The difference is that the HAOV (scattering angle in the pattern short-axis direction) increases slightly with the increase of OSB1 mass fraction, but, significantly increases with the increase of OSB2 mass fraction. All the results demonstrate that the smaller the particle size of OSB, the stronger the compensation for the light scattering of the pattern short-axis direction. That is to say, on the one hand, according to the Mie scattering theory, smaller particle size OSB can produce stronger scattering effects, the stronger scattering effect of OSB2 is responsible for its stronger compensation effect on the pattern short-axis scattering direction of the scattered pattern of the anisotropic light scattering material; on the other hand, smaller particle size OSB is more conducive to the production of spindle-shaped core-shell structures, and produce stronger multiple scattering effects, which all can better compensate the pattern short-axis direction light scattering.

### The influence of the draw ratio on the scattering angle

**Figure 8:**
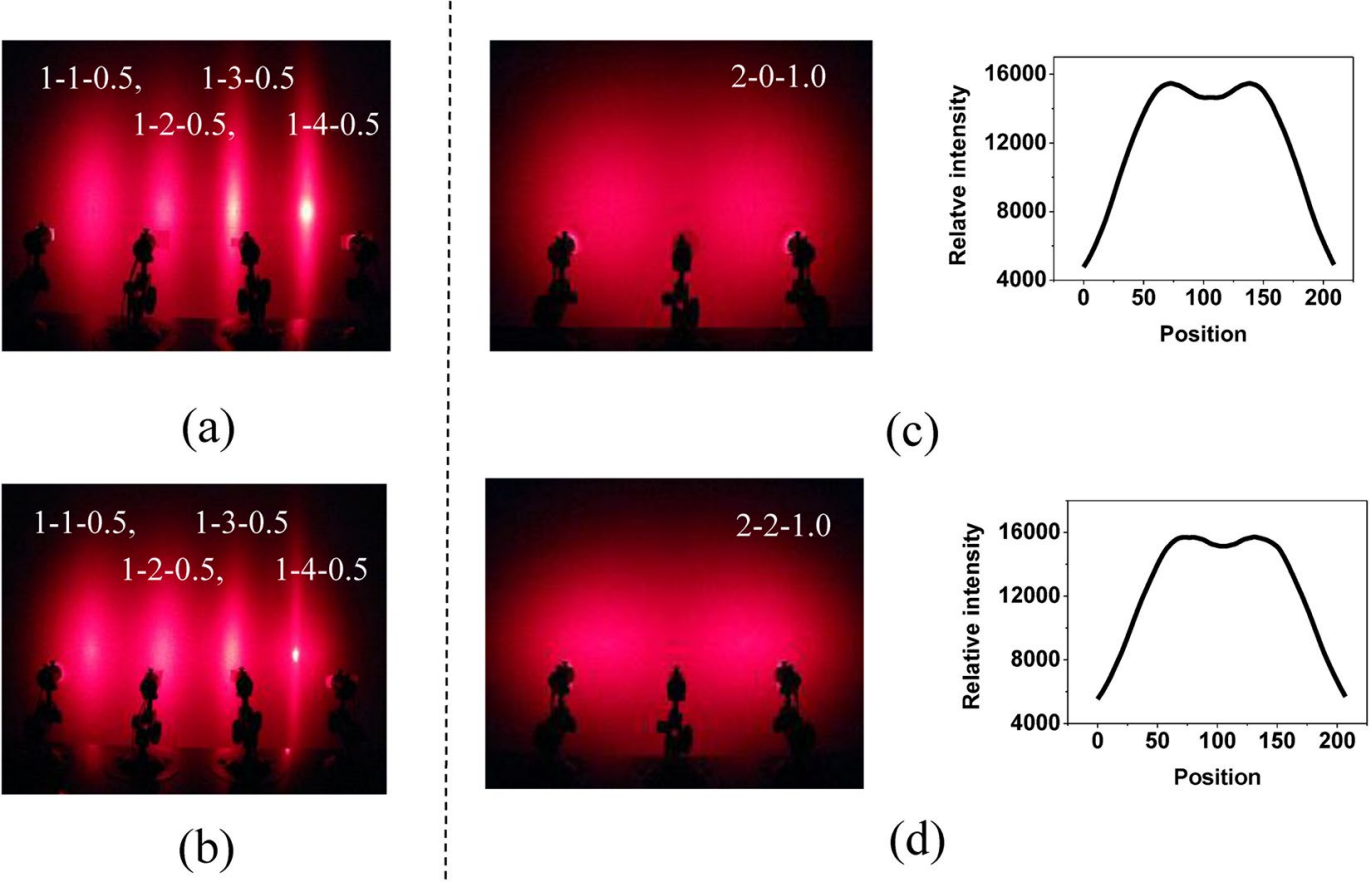
(**a**) and (**b**) The scattering patterns of the samples at different draw ratios when the OSB mass fraction is 0.5 wt%. (**c**) and (**d**) The two-source scattering effect of anisotropic and isotropic PC / SAN-OSB in the main scattering direction.

As shown in Fig. 5(b,c), the microscopic morphology of the samples with different draw ratios is very different, the draw ratio is increased, and the aspect ratio of the SAN phase in the sample is gradually increased. When the OSB mass fraction is 0.5 wt%, the light-scattering effects of the sample at different draw ratios are shown in Fig. 8(a,b), and the draw ratios from left to right are 1.75, 2.0, 2.25, and 2.5, respectively. With the increase of stretching ratio, the scattering angle in the pattern short-axis direction gradually decreases, the scattering range in the pattern long-axis direction slightly increases and the center brightness gradually increases. Compared with the OSB1 system, the OSB2 system has a significantly slower rate of scattering angle reduction and central light intensity increase in the pattern short-axis direction. However, when the draw ratio is increased to 2.5, the anisotropy and isotropic scattering of the OSB2 system have not matched, and a long light and circular pattern superimposed effect occurs. This indicates that in the case where the anisotropic particles and the isotropic particles are matched by light scattering, the smaller the OSB particle diameter, the smaller the loss in the pattern short-axis direction while increasing the light scattering in the pattern long-axis direction of the anisotropic light scattering. That is to say, smaller particle size OSB and smaller draw ratio are more conducive to the production of spindle-shaped core-shell structures. So they can produce stronger multiple scattering effects.

### Anisotropic and isotropic scattering contrast

The purpose of preparing anisotropic light-scattering materials is to distribute the incident light asymmetrically, mainly to meet the scattering light intensity in one direction and define this direction as the main scattering direction. Therefore, the scattering effect of the isotropic and anisotropic light-scattering materials in the main scattering direction when the two light sources are irradiated is compared. Figure 8(c,d) are a comparison of the scattering effects of 2-0-1.0 and 2-2-1.0 samples, respectively. It can be clearly seen that in the main scattering direction, the scattered light generated by the anisotropic light-scattering material is more uniform and the scattering curve is more gradual. The difference in relative light intensity of the peaks and troughs in the scattering curve can reflect the light-scattering ability of the light-scattering material in the main scattering direction. And the difference between the peak and the trough of the scattering curve produced by the anisotropic light-scattering material is remarkably smaller. Therefore, in the main scattering direction, more uniform scattered light and a larger scattering range and scattered light intensity are produced by the anisotropic light-scattering material.

## Conclusions

In this paper, a PC/SAN-OSB anisotropic light-scattering material containing novel spindle-shaped core-shell particles is prepared *in situ* by a thermal stretching method, which has excellent and easily tunable optical properties. Due to the better compatibility of OSB and SAN, OSB particles are selectively distributed in the SAN phase during melt processing to form a multiple scattering particle similar to the core-shell structure. The spherical particles gradually evolve into a spindle-shaped core-shell structure due to the orientation deformation of the shell-SAN phase during the hot stretching process. The influence of the size and mass fraction of the core (OSB particles) and the stretching ratio of the shell (SAN) on the anisotropic light-scattering effect was investigated. With the increase of OSB mass fraction, the center of scattered light pattern gradually decreased, HAOV and VAOV increased, but the HAOV increased significantly when smaller OSB was added. When the OSB particle size is larger, the effect of OSB mass fraction on the degree of light-scattering anisotropy is smaller. As the draw ratio increases, the scattering range in the pattern short-axis direction decreases with the increase of the stretching ratio and the scattering range in the pattern long-axis direction slightly increases. Anisotropic light-scattering materials can effectively improve the light intensity between the two light sources, and expand the scattering range and scattered light intensity of the light in the main scattering direction to achieve the purpose of rational use of light energy. In summary, on the one hand, according to the Mie scattering theory, smaller particle size OSB can produce stronger scattering effects; on the other hand, smaller particle size OSB and smaller draw ratio are more conducive to the production of spindle-shaped core-shell structures. The increase of OSB mass fraction is beneficial to the increase of the number of spindle-shaped core-shell structures, and they all produce stronger multiple scattering effects, which can improve the scattering range in the pattern short-axis direction while ensuring that the pattern long-axis direction light scattering range is not impaired.

## Data Availability

The datasets generated during and/or analysed during the current study are available from the corresponding author on reasonable request.
